# Supplementary material for: Comparing different farming habitats for mid-water rope nurseries to advance coral restoration efforts in the Maldives
Source: PeerJ. 2022 Feb 24;10:e12874. doi: 10.7717/peerj.12874 (PMC8882334; doi:10.7717/peerj.12874)
Supplement: Figure S1 — Donor colonies were collected as fragments of opportunity in 2018 and reared in two different nursery habitats on Athuruga. Colonies were fragmented and reciprocally stocked in 2020 in the lagoon and reef nursery at different depths. After a one-year monitoring period differences in fragment parameters such as survival, condition, growth and interactions with mutualists or predators were analysed. [file peerj-10-12874-s001.pdf]

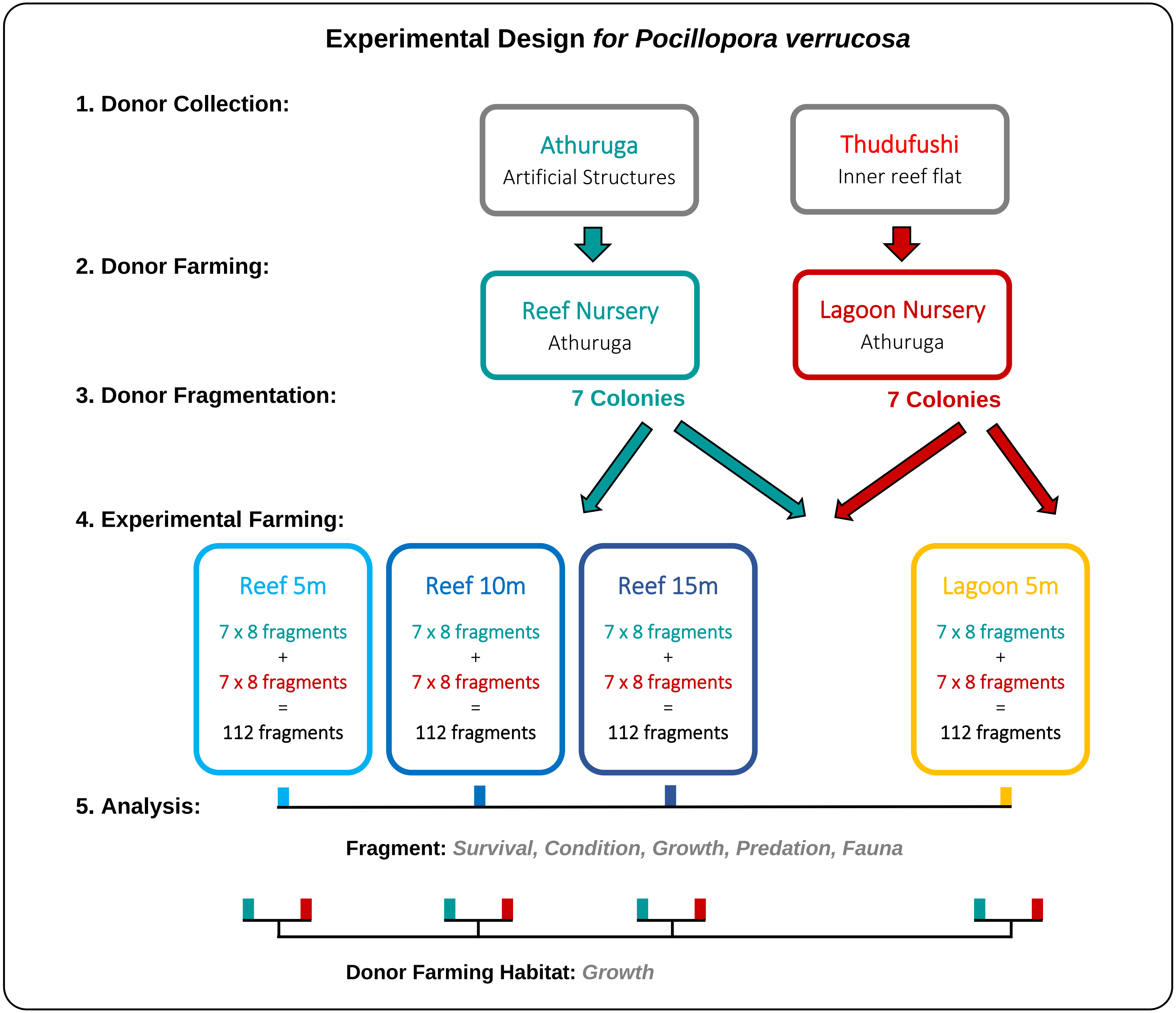
Experimental Design for Pocillopora verrucosa
1. Donor Collection:
Athuruga
Artificial Structures
Thudufushi
Inner reef flat
2. Donor Farming:
Reef Nursery
Athuruga
Lagoon Nursery
Athuruga
3. Donor Fragmentation:
7 Colonies
7 Colonies
4. Experimental Farming:
Reef 5m
7 x 8 fragments
+
7 x 8 fragments
=
112 fragments
Reef 10m
7 x 8 fragments
+
7 x 8 fragments
=
112 fragments
Reef 15m
7 x 8 fragments
+
7 x 8 fragments
=
112 fragments
Lagoon 5m
7 x 8 fragments
+
7 x 8 fragments
=
112 fragments
5. Analysis:
Fragment: Survival, Condition, Growth, Predation, Fauna
Donor Farming Habitat: Growth
